# Supplementary material for: Ultrafine nanoporous intermetallic catalysts by high-temperature liquid metal dealloying for electrochemical hydrogen production
Source: Nat Commun. 2022 Sep 2;13:5157. doi: 10.1038/s41467-022-32768-1 (PMC9440032; doi:10.1038/s41467-022-32768-1)
Supplement: Supplementary file 1 — Supplementary Information [file 41467_2022_32768_MOESM1_ESM.pdf]

Supplementary Information for

Ultrafine Nanoporous Intermetallic Catalysts by High-Temperature Liquid Metal Dealloying for Electrochemical Hydrogen Production

Ruirui Song[1,2], Jiuhui Han[3,4,5]*, Masayuki Okugawa[6,7], Rodion Belosludov[1], Takeshi Wada[1], Jing Jiang[1], Daixiu Wei[1], Akira Kudo[4], Yuan Tian[8], Mingwei Chen[8]* & Hidemi Kato[1]*

[1]Institute for Materials Research, Tohoku University, Sendai 980-8577, Japan

[2]Department of Materials Science, Graduate School of Engineering, Tohoku University, Sendai 980-8579, Japan

[3]Frontier Research Institute for Interdisciplinary Sciences (FRIS), Tohoku University, Sendai 980-8578, Japan

[4]WPI Advanced Institute for Materials Research, Tohoku University, Sendai 980-8577, Japan

[5]Tianjin Key Laboratory of Advanced Functional Porous Materials, Institute for New Energy Materials and Low-Carbon Technologies, Tianjin University of Technology, Tianjin 300384, China

[6]Division of Materials and Manufacturing Science, Graduate School of Engineering, Osaka University, 2-1 Yamadaoka, Suita, Osaka 565-0871, Japan

[7]Mathematics for Advanced Materials Open Innovation Laboratory, AIST, Sendai 980-8577, Japan

[8]Department of Materials Science and Engineering, Johns Hopkins University, Baltimore, MD 21218, USA

Correspondence and requests for materials should be addressed to Jiuhui Han (hanjh08@gmail.com), Mingwei Chen (mwchen@jhu.edu) or to Hidemi Kato (hikato@imr.tohoku.ac.jp)

Supplemental information including:

Supplementary Figure 1-23

Supplementary Table 1-5

Supplementary References

Supplementary Figures

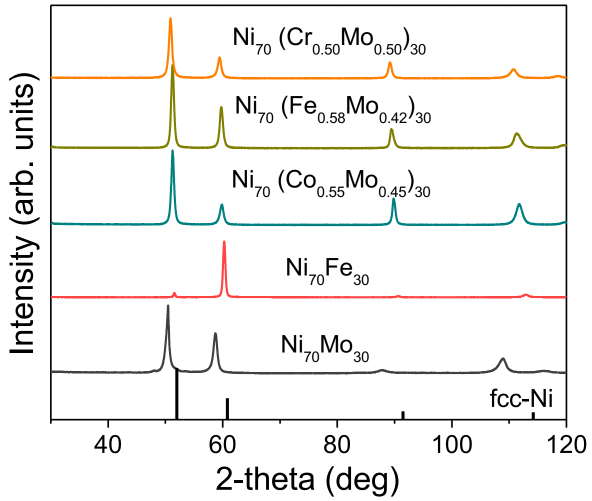

Supplementary Figure 1 XRD patterns for the Ni-Mo-M (M = Co, Fe, Cr), Ni-Mo and Ni-Fe precursor alloys. The standard pattern of Ni (fcc) (PDF#65-0380) is shown as the reference. All precursors are single phase fcc alloys. The peak shift is consistent with the relative atomic radius of the metals: Mo (139 pm) > Cr (128 pm) > Fe (126 pm) > Co (125 pm) > Ni (124 pm).

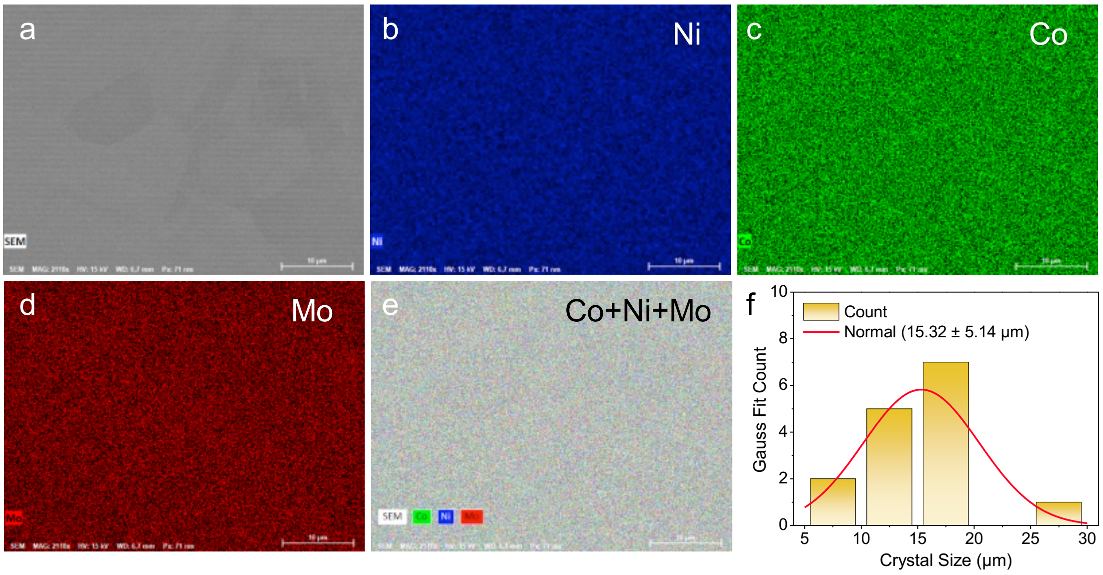

Supplementary Figure 2 Chemical composition and grain size of the $Ni_{70}(Co_{0.55}Mo_{0.45})_{30}$ precursor alloy. (a-e) SEM image and corresponding SEM-EDS elemental mappings of a polished surface of the $Ni_{70}(Co_{0.55}Mo_{0.45})_{30}$ precursor alloy. **(f)** Statistics for the grain size measured from **a**.

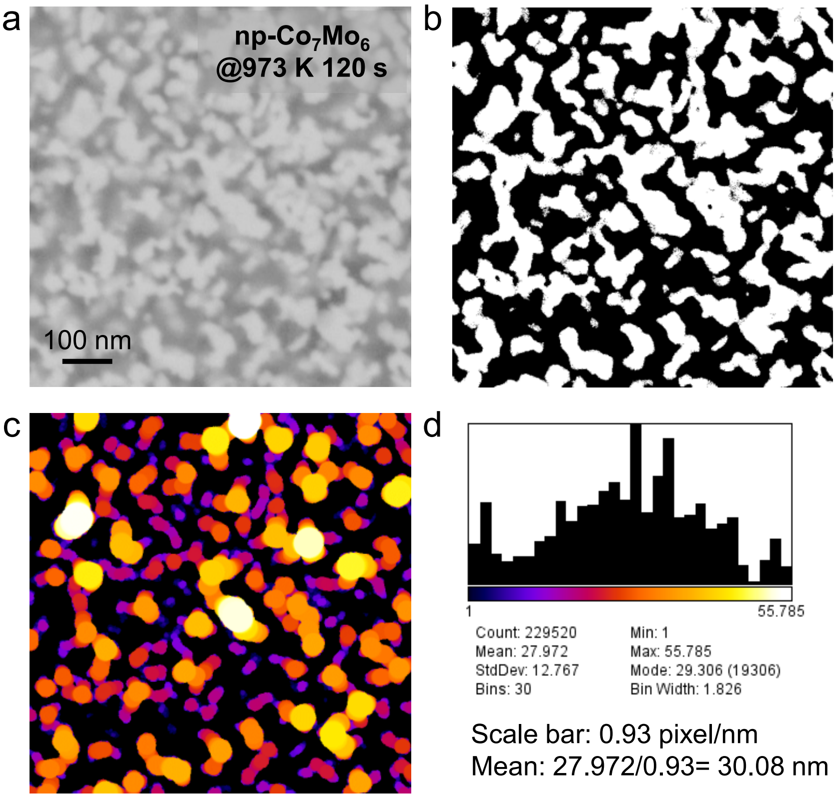

Supplementary Figure 3 Measurement of the ligament size using the Fiji package of the ImageJ software. (a) SEM image of ion-milled cross-section of np-Co_7Mo_6/Mg composite obtained after LMD at 973 K for 120s. The bright contrast is the μ-Co_7Mo_6 and the dark contrast is the hcp-Mg. **(b)** Binary image generated from the SEM image. **(c)** Local-thickness analysis. **(d)** Histogram result of the local-thickness revealing the mean ligament size.

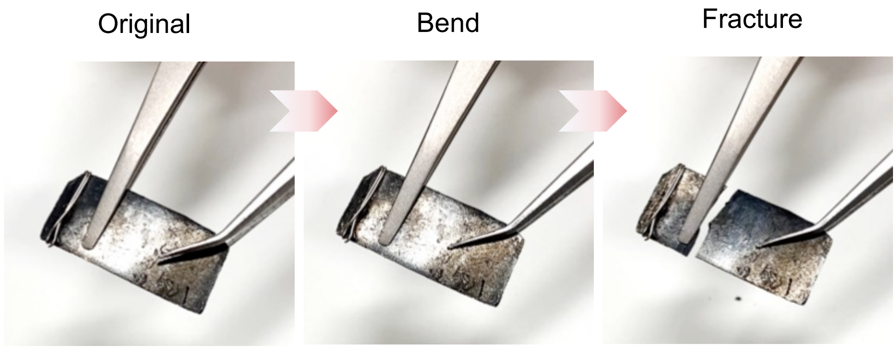

Supplementary Figure 4 Handling property test. Snapshots of a bending test showing the handling property and brittleness of free-standing np-Co_7Mo_6 sheet.

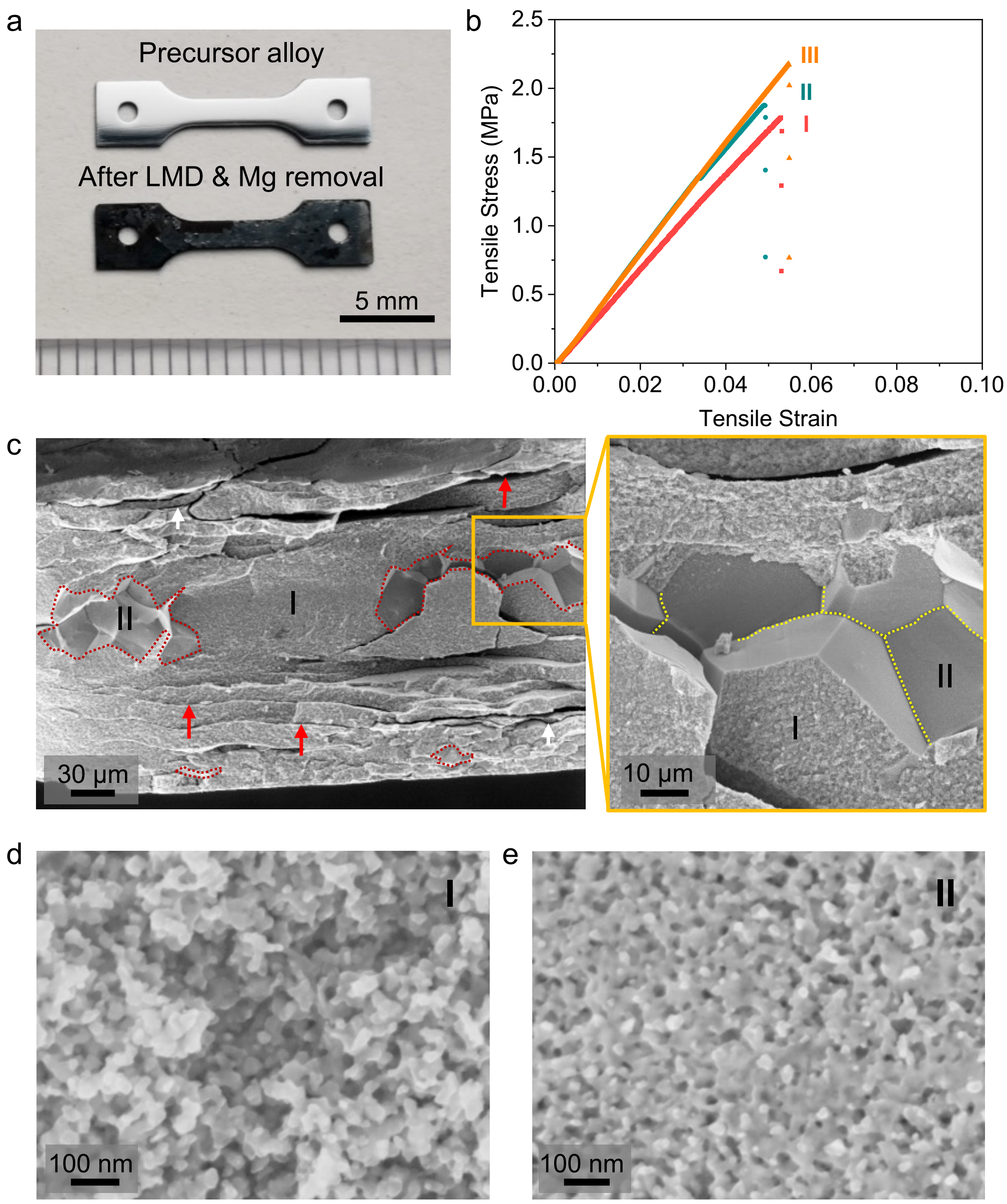

Supplementary Figure 5 Mechanical properties of the np-Co_7Mo_6. (a) Optical image of the precursor alloy and the np-Co_7Mo_6 sample for tensile test. The precursor alloy is cut into the dogbone shape with a gauge length of 3.8 mm, a width of 1.5 mm and a thickness of 200 μm. The macroscopic shape is well preserved after LMD and Mg removal with only slight thickness change from 200 μm to 170 μm due to volume contraction. **(b)** Tensile stress-strain curves of three np-Co_7Mo_6 samples. The fracture strength ranges from 1.8 to 2.2 MPa and the average value is ~2.0 MPa. **(c)** The fracture surface of np-Co_7Mo_6 after tensile test. The surface shows two different fracture zones: I, transgranular rough surfaces, and II, the flat region from intergranular brittle fracture (marked by red dotted lines). Laminar cracks formed during dealloying (marked by red arrows) can also be seen. **(d,e)** Zoom-in views of the fracture surfaces from region I and II, respectively.

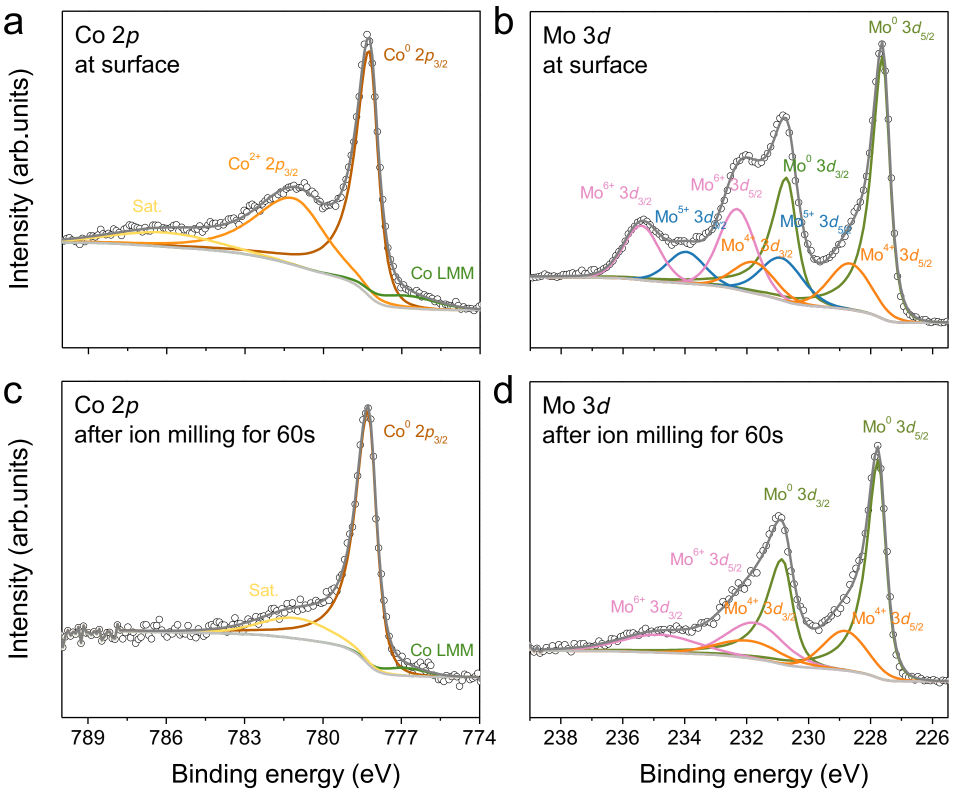

Supplementary Figure 6 XPS characterization of the np-Co_7Mo_6. XPS spectra for (**a**) Co 2*p* and (**b**) Mo 3*d* of the np-Co_7Mo_6 collected from the surface; XPS spectra for (**c**) Co 2*p* and (**d**) Mo 3*d* of the np-Co_7Mo_6 collected after ion milling for 60 seconds.

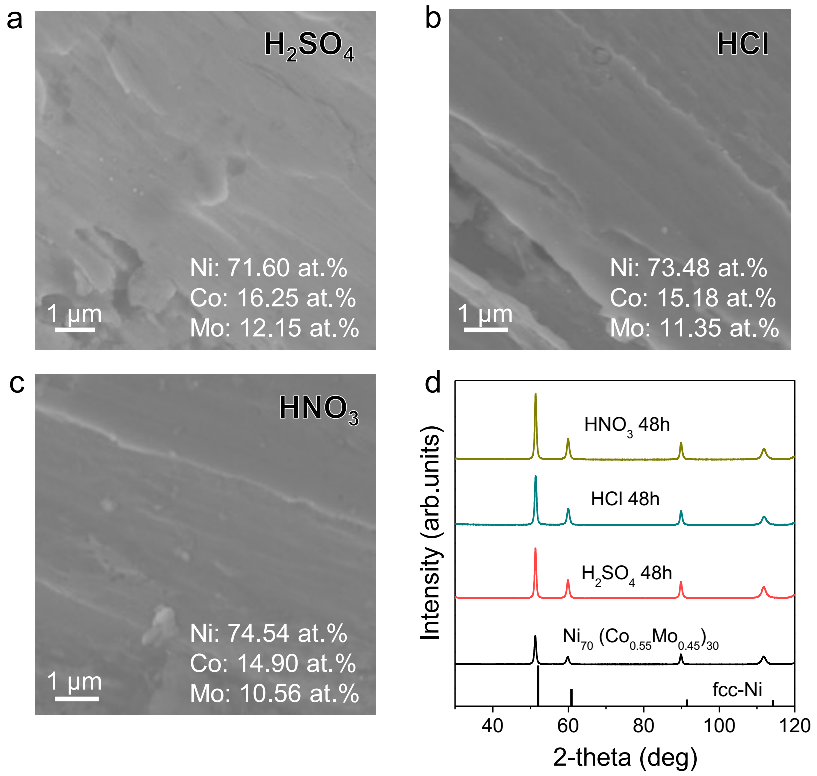

Supplementary Figure 7 Microstructural characterization of the $Ni_{70}(Co_{0.55}Mo_{0.45})_{30}$ alloy after chemical etching. SEM images of the surfaces of $Ni_{70}(Co_{0.55}Mo_{0.45})_{30}$ sheet after being immersed in **(a)** 0.5 M H_2SO_4, **(b)** 0.5 M HCl and **(c)** 0.5 M HNO_3 solution at room temperature for 48 h. The chemical compositions measured by SEM-EDS are also shown. **(d)** XRD patterns for the samples before and after chemical etching.

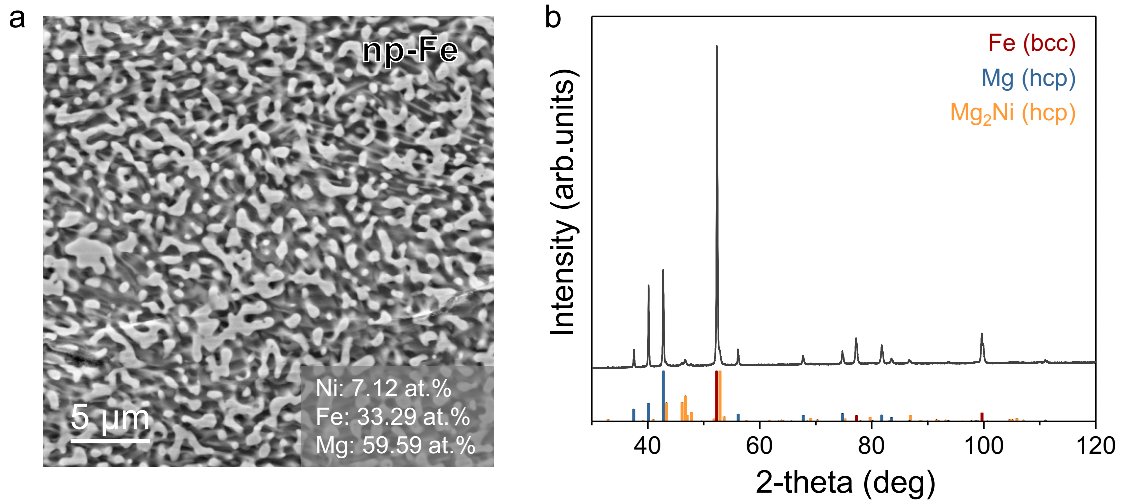

Supplementary Figure 8 Microstructural characterization of the np-Fe/Mg composite. (a) SEM image and **(b)** XRD pattern of the np-Fe/Mg composite obtained after LMD at 973 K for 120s. The standard patterns of bcc-Fe (PDF#65-4899), hcp-Mg (PDF#35-0821) and hcp-Mg_2Ni (PDF#75-1249) are also shown as references in **b**. In the SEM image, the bright contrast is the

bcc-Fe, the dark contrast is the hcp-Mg, and the laminar structure in the Mg phase with a light bright contrast is hcp-Mg_2Ni, which is formed from the alloying reaction between Mg melt and the dissolved Ni during cooling. The average feature/ligament size is 652.0 nm. This composite was not subjected to chemical etching to prepare nanoporous Fe as the active Fe cannot survive the chemical etching process.

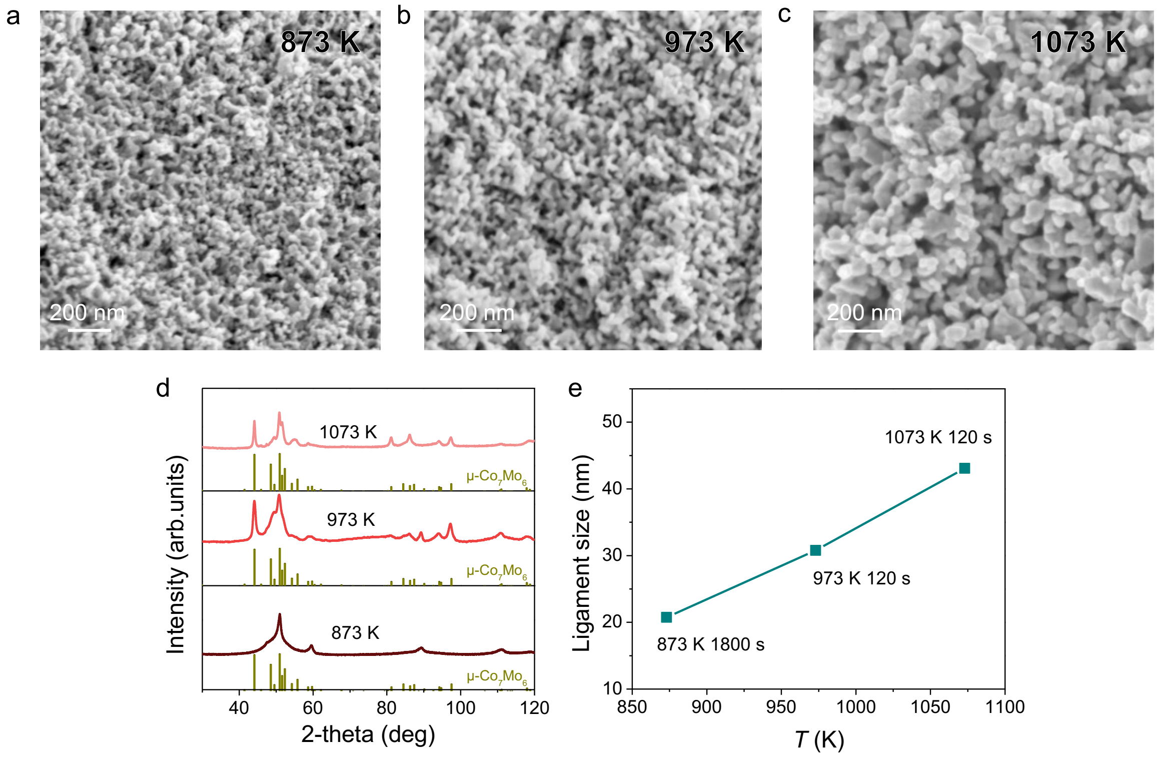

Supplementary Figure 9 Microstructural characterization of np-Co_7Mo_6 fabricated at different temperatures. (**a-c**) SEM images and (**d**) XRD patterns of np-Co_7Mo_6 fabricated at 873 K for 30 min, 973 K for 120s, and 1073 K for 120s. (**e**) The ligament size as a function of the dealloying temperature.

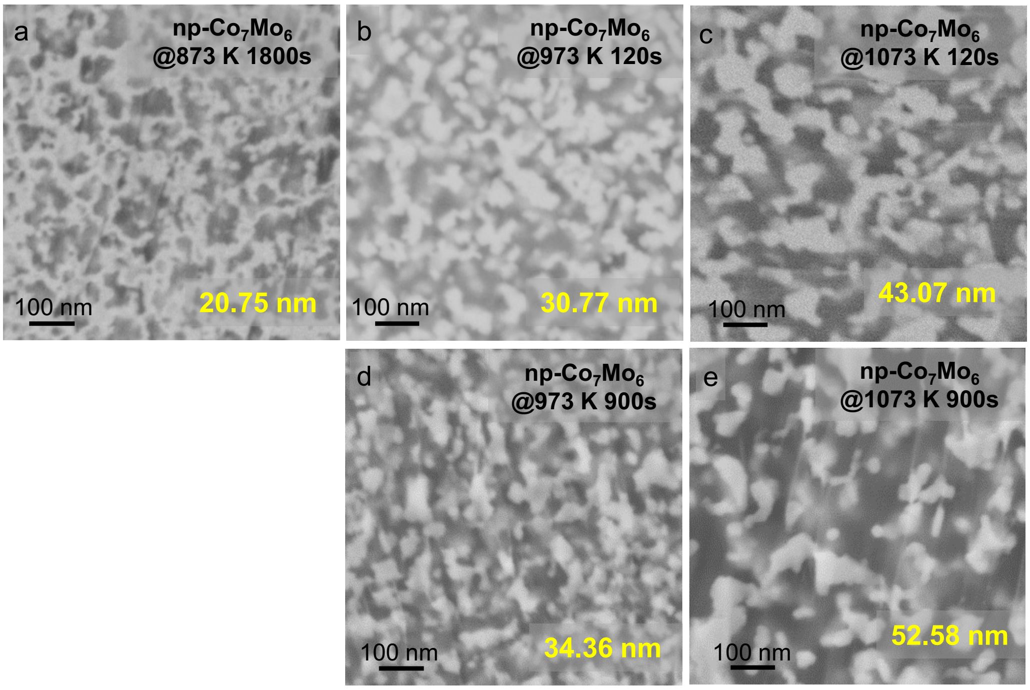

Supplementary Figure 10 SEM images of ion-milled cross-sections of np-Co_7Mo_6/Mg composite obtained after LMD at **(a)** 873 K for 1800s, **(b)** 973 K for 120s, **(c)** 1073 K for 120s, **(d)** 973 K for 900s, and **(e)** 1073 K for 900s. The bright contrast is the μ-Co_7Mo_6 and the dark contrast is the hcp-Mg. The average ligament sizes are given in yellow.

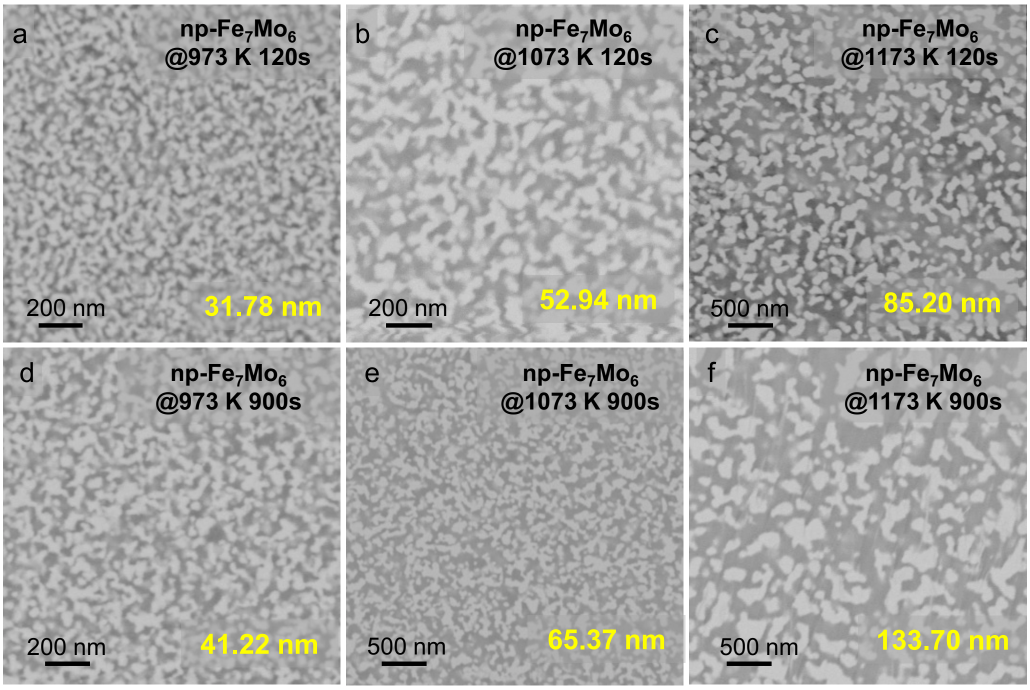

Supplementary Figure 11 SEM images of ion-milled cross-sections of np-Fe_7Mo_6/Mg composite obtained after LMD at **(a)** 973 K for 120s, **(b)** 1073 K for 120s, **(c)** 1173 K for 120s, **(d)** 973 K for 900s, **(e)** 1073 K for 900s, and **(f)** 1173 K for 900s. The bright contrast is

the μ-Co_7Mo_6 and the dark contrast is the hcp-Mg. The average ligament sizes are given in yellow.

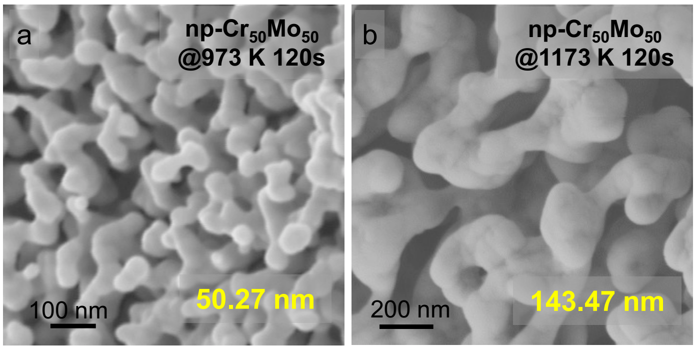

Supplementary Figure 12 SEM images of np-$Cr_{50}Mo_{50}$ fabricated by LMD at **(a)** 973 K for 120s, and **(b)** 1173 K for 120s. The average ligament sizes are given in yellow.

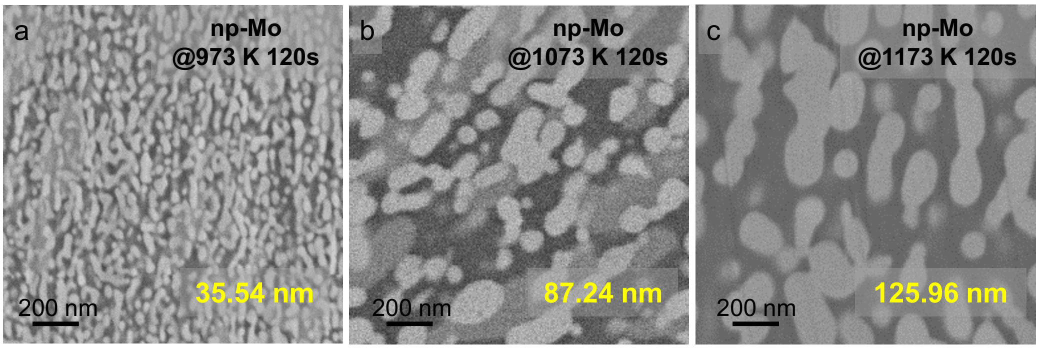

Supplementary Figure 13 SEM images of ion-milled cross-sections of np-Mo/Mg composite obtained after LMD at **(a)** 973 K for 120s, **(b)** 1073 K for 120s, and **(c)** 1173 K for 120s. The bright contrast is the bcc-Mo and the dark contrast is the hcp-Mg. The average ligament sizes are given in yellow.

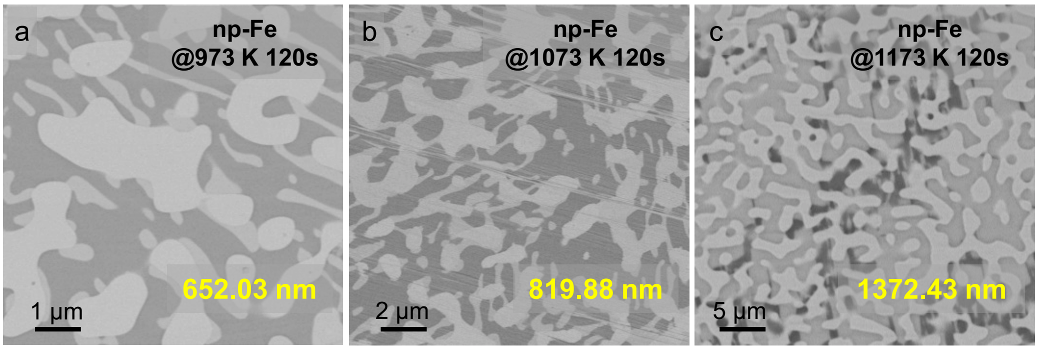

Supplementary Figure 14 SEM images of ion-milled cross-sections of np-Fe/Mg composite obtained after LMD at **(a)** 973 K for 120s, **(b)** 1073 K for 120s, and **(c)** 1173 K for 120s. The bright contrast is the bcc-Fe and the dark contrast is the hcp-Mg. The average ligament sizes are given in yellow.

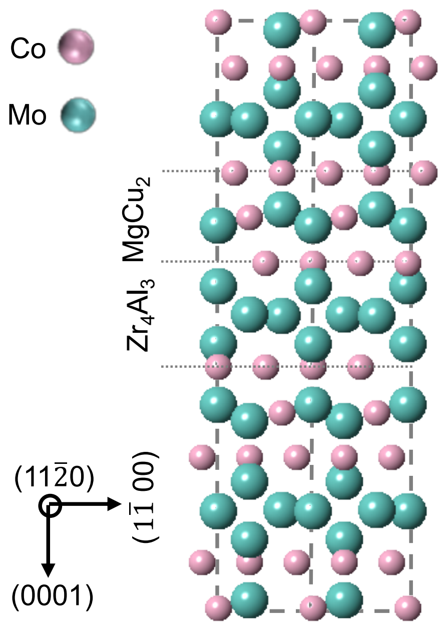

Supplementary Figure 15 Crystal structure of μ-Co_7Mo_6. Unit cell of the μ-Co_7Mo_6 structure visualized perpendicular to (11$\bar{2}$0) showing the alternating sub-cell stacking of Mg_2Cu-type Laves layers and Zr_4Al_3-layers.

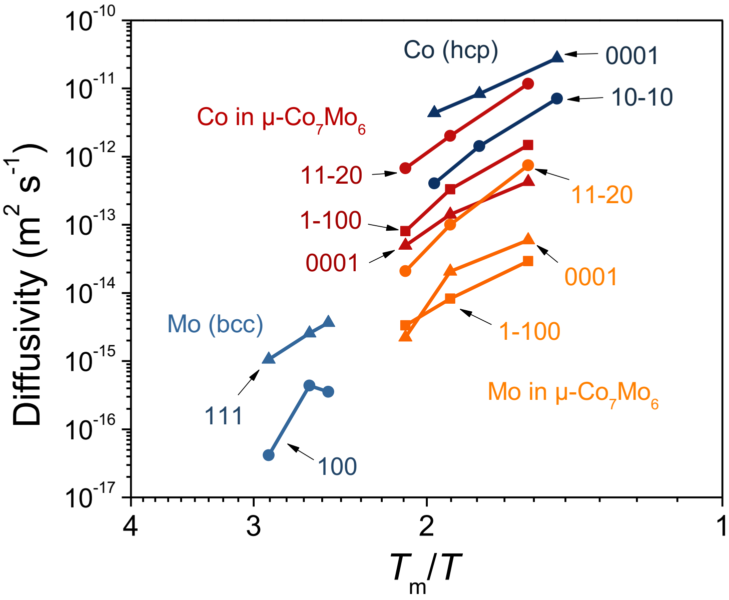

Supplementary Figure 16 Diffusivities calculated along differenct facets. Interfacial diffusivities along different facets of Mo (bcc), Co (hcp) and μ-Co_7Mo_6 at the temperatures of 900-1200 K.

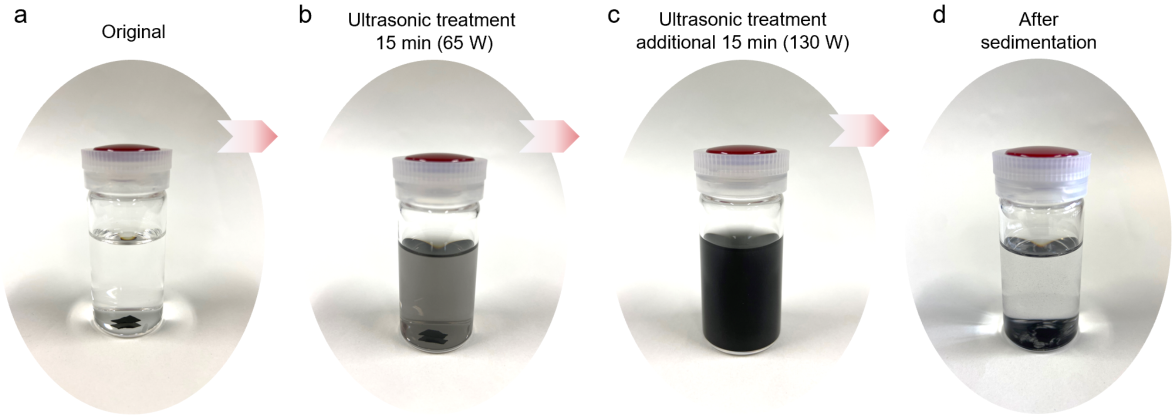

Supplementary Figure 17 Preparation of powder samples from np-Co_7Mo_6 sheets. Optical images of a free-standing np-Co_7Mo_6 sheet immersed in ethanol: **(a)** original state; **(b)** after ultrasonic treatment for 15 min at the low power of 65 W; **(c)** after ultrasonic treatment for an additional 15 min at the high power of 130 W; **(d)** after sedimentation. The free-standing np-Co_7Mo_6 sheet remains robust during the ultrasonication, but high-power ultrasonication can peel the sheet layer by layer to make powder samples. Therefore, a combination of mechanical grinding with a mortar and pestle and high-power ultrasonication at 130 W can be used to prepare powder samples from the as-dealloyed np-Co_7Mo_6 sheets.

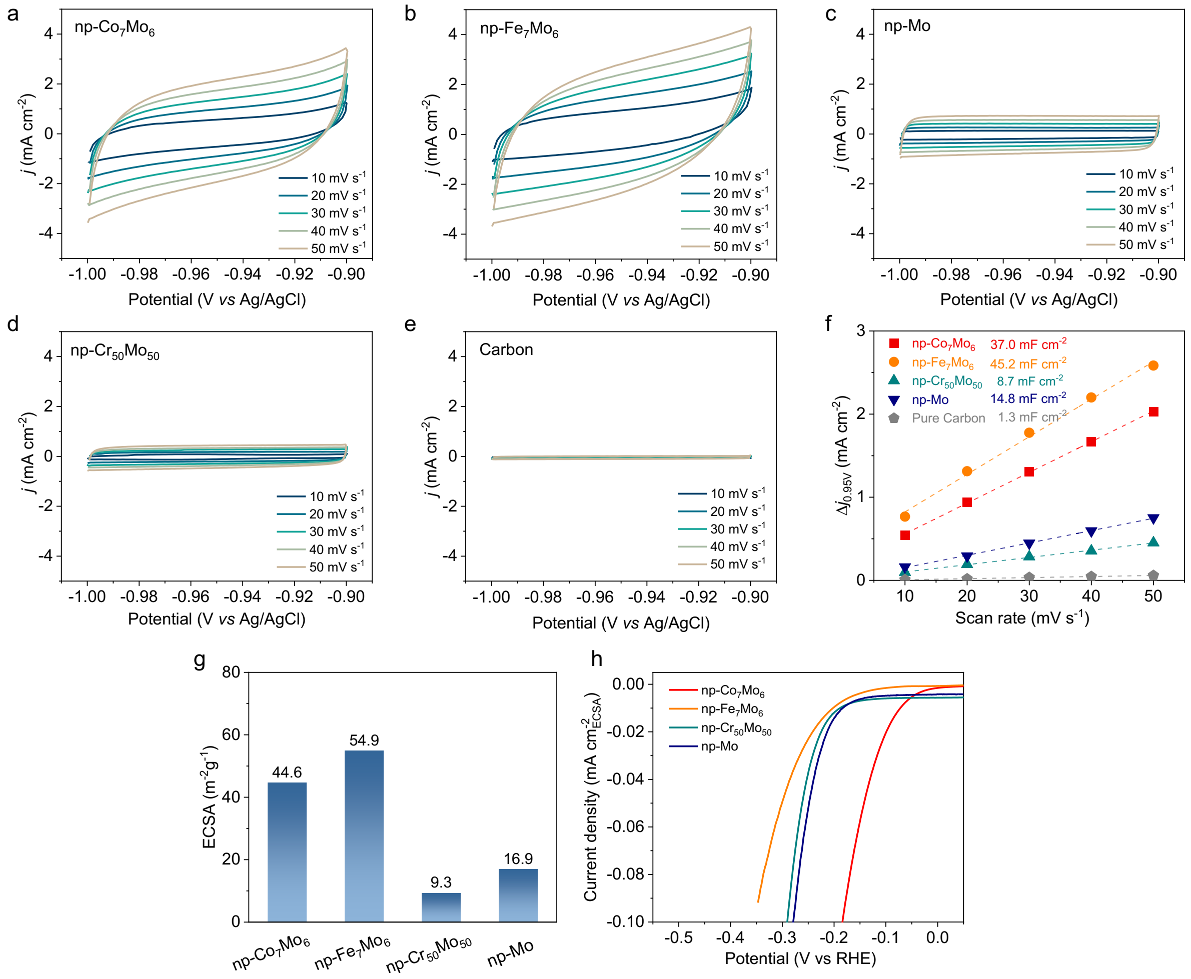

Supplementary Figure 18 Measurement of double layer capacitance and electrochemical active surface areas (ECSAs) for the powder samples. Cyclic voltammetry curves at the non-Faradaic potential region for **(a)** np-Co_7Mo_6, **(b)** np-Fe_7Mo_6, **(c)** np-$Cr_{50}Mo_{50}$, **(d)** np-Mo, and **(e)** pure carbon disk electrodes prepared from powder samples. **(f)** Current density difference ($\Delta j = |j_a - j_c|/2$) at -0.95 V plotted versus the scan rates. The values of double-layer capacitance are given. **(g)** ECSA of the various samples. The capacitance contribution from the carbon components in the mixture electrodes was subtracted for calculating the ECSA. **(h)** iR-corrected HER polarization curves with the current normalized by ECSA.

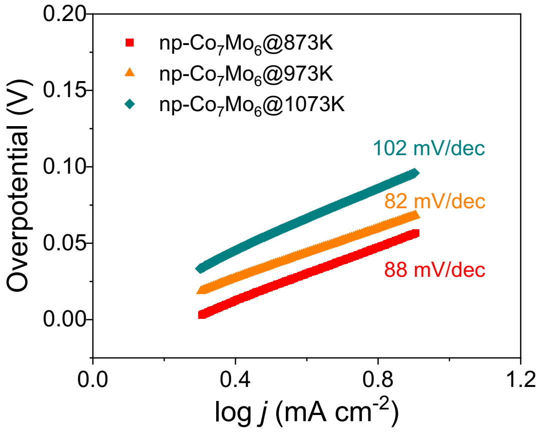

Supplementary Figure 19 Tafel plots for various np-Co_7Mo_6 samples. Tafel plots for np-Co_7Mo_6 fabricated by LMD at the temperatures of 873 K, 973 K, and 1073 K.

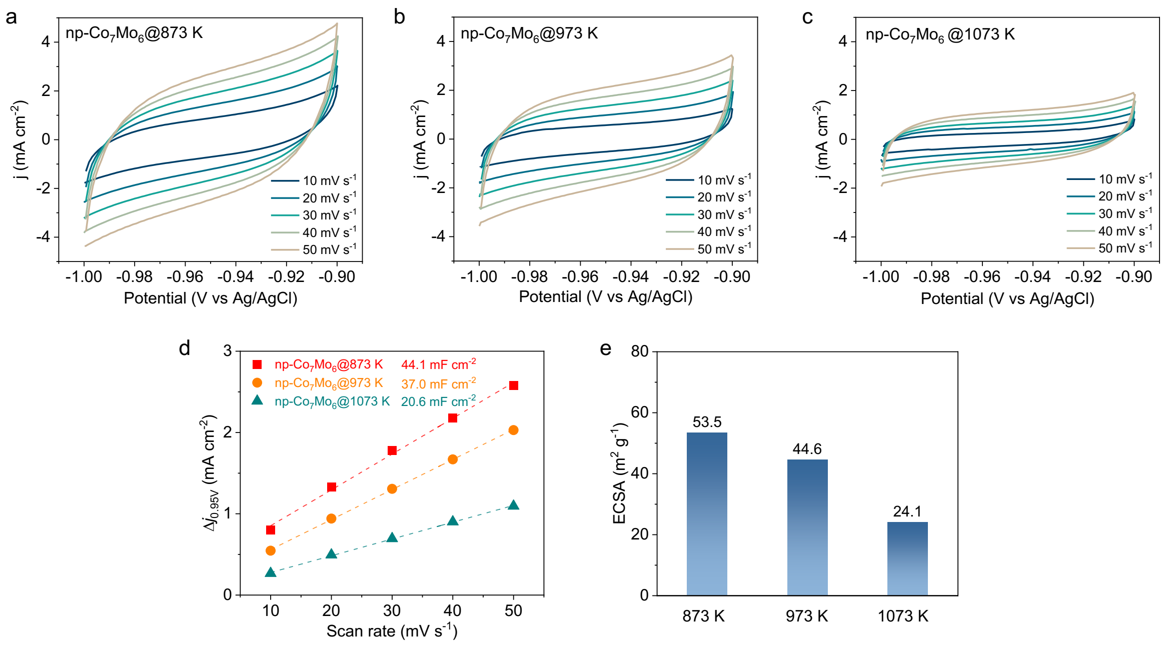

Supplementary Figure 20 Measurement of double layer capacitance and electrochemical active surface areas (ECSAs) for various np-Co_7Mo_6 samples. Cyclic voltammetry curves at the non-Faradaic potential region for np-Co_7Mo_6 fabricated at **(a)** 863 K, **(b)** 973 K, and **(c)** 1073K. The data were collected from disk electrodes prepared from powder samples. **(d)** Current density difference ($\Delta j = |j_a - j_c|/2$) at -0.95 V plotted versus the scan rates. The values of double-layer capacitance are given. **(e)** ECSA of the various samples. The capacitance contribution from the carbon components in the mixture electrodes was subtracted for calculating the ECSA.

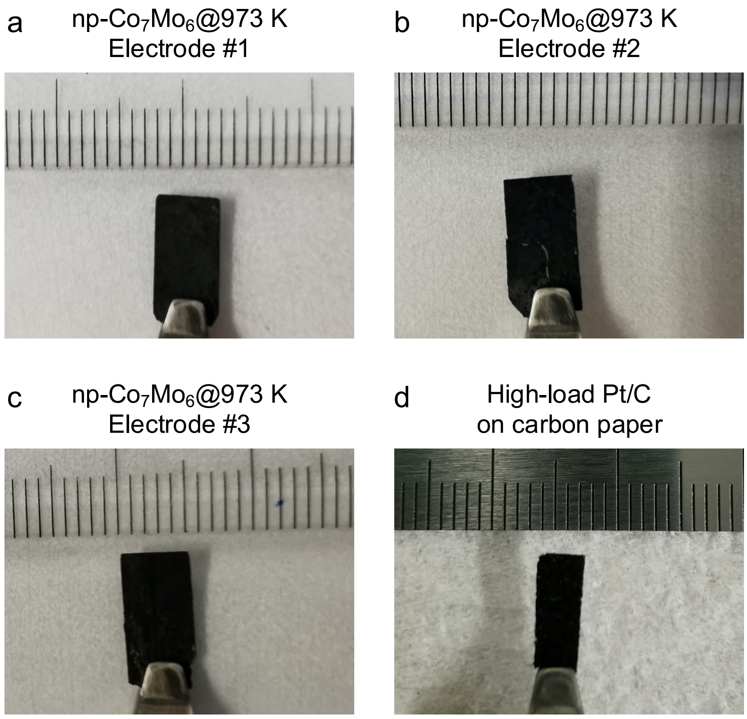

Supplementary Figure 21 Macroscopic morphology of the electrodes. Optical images of **(a-c)** three batches of self-supported np-Co_7Mo_6 sheet electrodes with a dimension of 1 cm × 0.5 cm × 170 μm fabricated by the same procedure, and **(d)** a high-load Pt/C electrode.

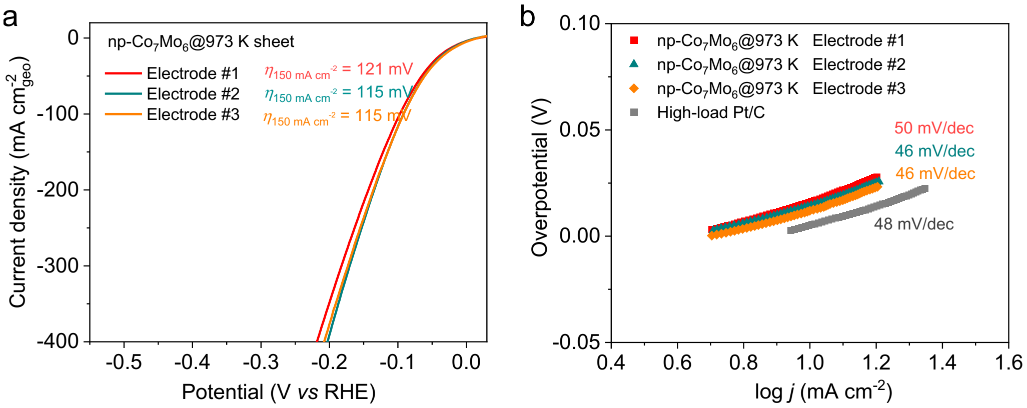

Supplementary Figure 22 Reproducibility tests. (a) iR-corrected HER polarization curves of three batches of self-supported np-Co_7Mo_6 sheet electrodes. **(b)** Tafel plots of the three self-supported np-Co_7Mo_6 sheet electrodes and a high-load Pt/C electrode.

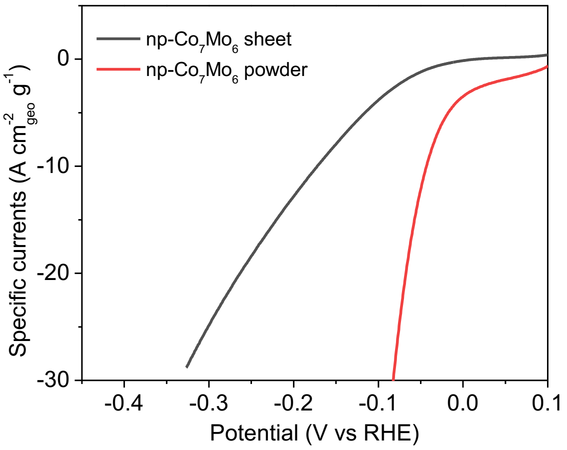

Supplementary Figure 23 Comparison of the material utilization efficiency. iR-corrected HER polarization curves of np-Co_7Mo_6 sheet and powder electrodes with the current normalized by the geometric area and the mass of electrocatalyst, showing the higher materials utilization efficiency of the powder electrode than the self-supported sheet electrode.

Supplementary Tables

Supplementary Table 1 Chemical compositions (at.%) of the precursor alloys and the dealloyed np-M-Mo (M = Co, Fe, Cr) samples prepared at different temperatures by LMD.

	**Sample**	**Ni**	**Co**	**Fe**	**Cr**	**Mo**	**Mg**
**Before LMD**	$Ni_{70}Mo_{30}$	71.20	-	-	-	28.80	-
	$Ni_{70}Fe_{30}$	69.58	-	30.42	-	-	-
	$Ni_{70}(Co_{0.55}Mo_{0.45})_{30}$	69.30	16.80	-	-	13.90	-
	$Ni_{70}(Fe_{0.58}Mo_{0.42})_{30}$	69.90	-	17.25	-	12.85	-
	$Ni_{70}(Cr_{0.50}Mo_{0.50})_{30}$	70.27	-	-	15.05	14.68	-
**After LMD**	np-Mo (973 K)	0.56	-	-	-	99.42	0.01
	np-Co_7Mo_6 (873 K)	7.69	46.01	-	-	46.30	0
	np-Co_7Mo_6 (973 K)	4.30	50.20	-	-	44.25	1.25
	np-Co_7Mo_6 (1073 K)	4.63	47.67	-	-	46.86	0.84
	np-Fe_7Mo_6 (973 K)	2.12	-	54.42	-	43.11	0.34
	np-$Cr_{50}Mo_{50}$ (973 K)	0.38	-	-	49.68	49.92	0.02
	np-$Cr_{50}Mo_{50}$ (1173 K)	0.74	-	-	49.89	49.38	0

Supplementary Table 2 Unit cell parameters of fcc-$Ni_{70}(Co_{0.55}Mo_{0.45})_{30}$ and μ-Co_7Mo_6.

	**Crystal System**	**Space group**	***a*** **(Å)**	***b*** **(Å)**	***c*** **(Å)**
$Ni_{70}(Co_{0.55}Mo_{0.45})_{30}$	Cubic	Fm-3m	3.585	3.585	3.585
μ-Co_7Mo_6	Trigonal	R-3m	4.762	4.762	25.617

Supplementary Table 3 Activation energy for interfacial diffusion of Co (hcp), Mo (bcc) and Co, Mo in μ-Co_7Mo_6, calculated according to $D = D_0 \exp(-E_a/kT)$ on basis of the temperature-dependent average interfacial diffusivities obtained from molecular dynamics simulations.

Atom	Activation energy of interfacial diffusion (kJ mol^{-1})
Co (hcp)	59.8
Mo (bcc)	84.2
Co in μ-Co_7Mo_6	84.7
Mo in μ-Co_7Mo_6	102.5

Supplementary Table 4 Comparison of the basic materials properties and electrochemical performance of the nanoporous intermetallic compounds tested as powder samples.

Sample	Ligament size (nm)	ECSA (m^2 g^{-1})	R_{ct} (Ω)	$\eta_{@10\ mAcm-2}$ (mV)	Tafel slope (mV dec^{-1})
np-Co_7Mo_6 (873 K)	20.7	53.5	6.40	66	88
np-Co_7Mo_6 (973 K)	30.8	44.6	5.13	76	82
np-Co_7Mo_6 (1073 K)	43.1	24.1	9.41	106	102
np-Fe_7Mo_6 (973 K)	31.8	54.9	75.99	197	124
np-$Cr_{50}Mo_{50}$ (973 K)	50.3	9.3	309.7	265	110
np-Mo (973 K)	35.5	16.9	90.23	228	120

Supplementary Table 5 Comparison of HER performance of the electrocatalysts in KOH aqueous solution.

Electrocatalysts	$\eta_{@10\ mAcm^{-2}}$ (mV)	Tafel slope ($mVdec^{-1}$)	Refs.
np-Co_7Mo_6 sheet	14	46	This work
Mo-doped NiCu	95	41	1
NiMo film	157	60	2
Ni_4Mo alloy nanosheets	32.2	37	3
$CoNiO_2/Ni_{0.46}Co_{0.54}$	58	36.4	4
$MoNi_4/MoO_2$@Ni	15	30	5
Ni_4Mo/GNS	69	66.4	6
Co_3Mo NP@Mo oxide array	68	61.3	7
Al-$NiMoO_4$-rods	131	141	8
Ni-Mo nanotube	44	55	9
Co_3Mo/np-Cu	28	32	10
np-NiMnFeMo alloy	12	40	11
CoMoMg nanomesh	36	165	12
$Co_1Fe_1Mo_{1.8}O$ NMs@NF	210	32	13
Fe-MoO_2/MoO_3/ENF	36	84.2	14
np-$Cu_{100-x}Mo_x$	97	110	15
Co-Mo-Cu coatings	119	82	16
Co-Mo-P@C	159	131	17
$CoMoN_x$ NSAs/NF	91	70.3	18
Co/MoN nanoflake	52	77.5	19
$CoMo_3$ nano-heterostructure	165	57	20

Supplementary References

1 Wang, Z. *et al.* Mo-doped NiCu as an efficient and stable electrocatalyst for the hydrogen evolution reaction. *New J. Chem.* **43**, 9652-9657 (2019).

2 Pehlivan, İ. B., Arvizu, M. A., Qiu, Z., Niklasson, G. A. & Edvinsson, T. Impedance spectroscopy modeling of nickel-molybdenum alloys on porous and flat substrates for applications in water splitting. *J. Phys. Chem. C* **123**, 23890-23897 (2019).

3 Xie, Z., Zou, Y., Deng, L. & Jiang, J. Self-supporting Ni-M (M = Mo, Ge, Sn) alloy nanosheets via topotactic transformation of oxometallate intercalated layered nickel hydroxide salts: synthesis and application for electrocatalytic hydrogen evolution reaction. *Adv. Mater. Interfaces* **7**, 1901949 (2020).

4 Zhou, K., Zhang, Q., Liu, J., Wang, H. & Zhang, Y. Crystal phase tuning and valence engineering in non-noble catalysts for outstanding overall water splitting. *J. Mater. Chem. A* **8**, 4524-4532 (2020).

5 Zhang, J. *et al.* Efficient hydrogen production on $MoNi_4$ electrocatalysts with fast water dissociation kinetics. *Nat. Commun.* **8**, 1-8 (2017).

6 Zhou, Y. *et al.* Mechanistic study on nickel-molybdenum based electrocatalysts for the hydrogen evolution reaction. *J. Catal.* **388**, 122-129 (2020).

7 Chen, J. *et al.* Nesting Co_3Mo binary alloy nanoparticles onto molybdenum oxide nanosheet arrays for superior hydrogen evolution reaction. *ACS Appl. Mater. Interfaces* **11**, 9002-9010 (2019).

8 Ma, L., Liu, Z., Chen, T., Liu, Y. & Fang, G. Aluminum doped nickel-molybdenum oxide for both hydrogen and oxygen evolution reactions. *Electrochim. Acta* **355**, 136777 (2020).

9 Zhang, J.-Y. *et al.* Energy-saving hydrogen production coupling urea oxidation over a bifunctional nickel-molybdenum nanotube array. *Nano Energy* **60**, 894-902 (2019).

10 Shi, H. *et al.* Spontaneously separated intermetallic Co_3Mo from nanoporous copper as versatile electrocatalysts for highly efficient water splitting. *Nat. Commun.* **11**, 1-10 (2020).

11 Liu, H. *et al.* Free-standing nanoporous NiMnFeMo alloy: an efficient non-precious metal electrocatalyst for water splitting. *Chem. Eng. J.* **404**, 126530 (2021).

12 Pei, L. *et al.* Three-dimensional CoMoMg nanomesh based on the nanoscale Kirkendall effect for the efficient hydrogen evolution reaction. *J. Alloys Compd.* **857**, 158086 (2021).

13 Pei, L. *et al.* Mo-doping induced edge-rich cobalt iron oxide ultrathin nanomeshes as efficient bifunctional electrocatalysts for overall water splitting. *Electrochim. Acta* **368**, 137651 (2021).

14 Chen, J. *et al.* High-performance bifunctional Fe-doped molybdenum oxide-based electrocatalysts with in situ grown epitaxial heterojunctions for overall water splitting. *Int. J. Hydrogen Energy* **45**, 24828-24839 (2020).

15 Luo, M. *et al.* Dilute molybdenum atoms embedded in hierarchical nanoporous copper accelerate the hydrogen evolution reaction. *Scr. Mater.* **191**, 56-61 (2021).

16 Santos, H. L. S., Corradini, P. G., Medina, M. & Mascaro, L. H. Effect of copper addition on cobalt-molybdenum electrodeposited coatings for the hydrogen evolution reaction in alkaline medium. *Int. J. Hydrogen Energy* **45**, 33586-33597 (2020).

17 Shi, J. *et al.* Cobalt-molybdenum bimetal phosphides encapsulated in carbon as efficient and durable electrocatalyst for hydrogen evolution. *ChemistrySelect* **5**, 14312-14319 (2020).

18 Lu, Y. *et al.* Bimetallic Co-Mo nitride nanosheet arrays as high-performance bifunctional electrocatalysts for overall water splitting. *Chem. Eng. J.* **411**, 128433 (2021).

19 Sun, J. *et al.* Co/MoN hetero-interface nanoflake array with enhanced water dissociation capability achieves the Pt-like hydrogen evolution catalytic performance. *Appl. Catal., B* **286**, 119882 (2021).

20 Jeghan, S. M. N., Kim, J. & Lee, G. Hierarchically designed CoMo marigold flower-like 3D nano-heterostructure as an efficient electrocatalyst for oxygen and hydrogen evolution reactions. *Appl. Surf. Sci.* **546**, 149072 (2021).
