# Supplementary material for: Ultrafine nanoporous intermetallic catalysts by high-temperature liquid metal dealloying for electrochemical hydrogen production
Source: Nat Commun. 2022 Sep 2;13:5157. doi: 10.1038/s41467-022-32768-1 (PMC9440032; doi:10.1038/s41467-022-32768-1)
Supplement: Supplementary file 3 — Description of Additional Supplementary Files [file 41467_2022_32768_MOESM3_ESM.pdf]

Description of Additional Supplementary Files

File Name: Supplementary Movie 1
Description: The bending test of nanoporous μ-Co7Mo6 sheet sample.
